# Coronary Microvascular Dysfunction and the Role of Noninvasive Cardiovascular Imaging

**DOI:** 10.3390/diagnostics10090679

**Published:** 2020-09-09

**Authors:** Muhammad Talha Ayub, Dinesh Kalra

**Affiliations:** Division of Cardiology, Rush University Medical Center, Chicago, IL 60612, USA; muhammad_talha_ayub@rush.edu

**Keywords:** coronary microvascular dysfunction, angina, noninvasive coronary imaging

## Abstract

Patients with coronary microvascular dysfunction (CMD) have significantly higher rates of cardiovascular events, including hospitalization for heart failure, sudden cardiac death, and myocardial infarction (MI). In CMD, several pathophysiological changes lead to functional and structural abnormalities in the coronary microvasculature, which disrupt the ability of the vessels to vasodilate and augment myocardial blood flow in response to increased myocardial oxygen demand, causing ischemia and angina. With the advent of more advanced non-invasive cardiac imaging techniques, the coronary microvasculature has been subjected to more intense study in the past two decades—this has led to further insights into the diagnosis, pathophysiology, treatment, prognosis and follow-up of CMD. This review will highlight and compare the salient features of the currently available non-invasive imaging modalities used in these patients, and discuss the clinical utility of these techniques in the workup and management of these patients.

## 1. Introduction

In the past 10 to 15 years, coronary microvascular dysfunction (CMD) has gained more recognition and is being increasingly diagnosed as a cause of angina and morbidity in patients who have objective evidence of myocardial ischemia but do not have obstructive coronary artery disease (CAD) on angiography. These patients often experience “microvascular angina” [1,2], which is as debilitating as patients who have obstructive CAD. Their prognosis is also not benign as previously believed, and they have higher rates of cardiovascular events, including myocardial infarction (MI), hospitalization for unstable angina, heart failure and sudden cardiac death, as well as a reduced quality of life [3,4,5]. Diagnostic testing, either in the cardiac catheterization laboratory, or via noninvasive imaging modalities, is useful to confirm the diagnosis, improve risk stratification, guide therapy and provide objective disease monitoring tools in these patients.

## 2. Overview of the Coronary Microvascular Circulation

### 2.1. Anatomic and Physiological Considerations

**Figure 1:**
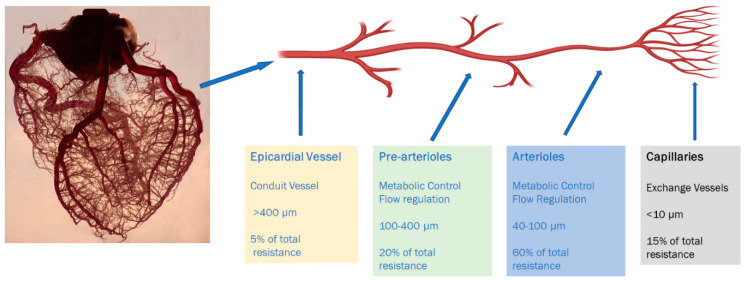
Anatomical and functional classification of the coronary macro and micro-arterial system.

The coronary microvasculature represents the distal-most compartment of the coronary arterial system and comprises intramural arterioles with diameters of less than 100 μm (Figure 1) [6]. Their role is to match myocardial blood supply and oxygen consumption to demand by regulating coronary blood flow. This is achieved by coordinating the vascular resistance within the different arterioles and smaller, distal vessels in the coronary arterial tree in response to the release of metabolites by the myocardium, which occurs when there is an increase in myocardial oxygen consumption [7,8].

Coronary microvascular function is a crucial determinant of perfusion. Coronary autoregulatory mechanisms maintain a relatively constant coronary perfusion despite wide range of variations in perfusion pressures [9,10]. This is achieved through changes in coronary resistance. Coronary flow decreases significantly, causing myocardial ischemia, once perfusion pressure falls below the accepted range. The complex control of the resistance in the coronary arterial tree and microcirculation involves shear-mediated neurohormonal regulation, metabolic regulation, and myogenic control [6].

### 2.2. Pathogenesis

CMD results from heightened sensitivity of the coronary microcirculation to vasoconstrictor stimuli associated with a limited microvascular vasodilator capacity. This manifests as suboptimal coronary vasodilator response to exercise or pharmacological stress, which can be measured by various invasive and noninvasive techniques [3,7,11,12]. The role of microvascular inflammation as a trigger of CMD has been a subject of research in the field of preventive cardiology—there is significant evidence that microvascular dysfunction is associated with increased systemic inflammation, and may precede or coexist with coronary atherosclerosis [10]. Patients with systemic inflammatory conditions, such as systemic lupus erythematosus or rheumatoid arthritis found to have nonobstructive coronary artery disease (CAD) by angiography, often demonstrate impaired coronary flow reserve (CFR), which is directly related to the disease duration [9,13,14].

Many patients with CMD also have macrovascular atherosclerosis, albeit usually nonobstructive disease [15]. CMD plays an important role in the pathogenesis of several cardiovascular syndromes such as HFpEF, and the cardiomyopathies associated with stress, obesity and diabetes [16,17].

## 3. Prognosis and Outcomes

Patients with CMD do not have a benign prognosis as was traditionally believed. They have a higher incidence of cardiovascular events, including hospitalization for heart failure, sudden cardiac death, and MI as compared to age-matched controls, irrespective of gender [18,19,20,21,22] Older age, hypertension, diabetes, and smoking have been associated with increased mortality, whereas hyperlipidemia, family history of premature CAD, or pretest CAD likelihood do not impact the prognosis significantly [2]. The risk of adverse outcomes is higher in women, in whom angina first occurs in the perimenopausal period. In a systematic review of 1694 patients with CMD by Vermeltfoort et al., the rate of MI, revascularization or death at 5 years was 1.5%, and rate of overall cardiac events (including new CAD, heart failure and cardiovascular events) was 4.8% [23]. In the WISE study, women without obstructive CAD but with evidence of myocardial ischemia (diagnosed by abnormal magnetic resonance spectroscopy) were similarly noted to have more adverse cardiovascular outcomes, notably higher rates of hospitalization with unstable angina, repeat catheterization, and greater treatment costs [1].

## 4. Clinical Variants of CMD

CMD can be differentiated into three major categories: [6,12]

A. Primary CMD:

Microvascular dysfunction in the absence of imaging and clinical evidence of obstructive CAD or myocardial disease constitutes this category. It can be a precursor of CAD and/or can result from vasomotor instability secondary to genetic or acquired cardiovascular risk factors. This condition has also been variably termed microvascular angina or syndrome X [24].

B. Secondary CMD:
  This is further classified in to two categories.
  (i) CMD in the presence of obstructive CAD. Microvascular dysfunction can concomitantly exist with stable and unstable CAD.
  (ii) CMD in the presence of myocardial disease. Microvascular dysfunction can occur secondary to arterial remodeling, intimal hypertrophy along with interstitial and perivascular fibrosis resulting from various cardiomyopathies.
C. Iatrogenic CMD:

Coronary microcirculation can become dysfunctional subsequent to a percutaneous coronary intervention that can lead to vasoconstrictor response and/or distal embolization caused at the time of revascularization.

## 5. Assessment of Microvascular Blood Flow

Direct assessment of microvascular flow is complex as no technique allows direct visualization of the coronary microcirculation. Microvascular perfusion cannot be directly assessed through coronary blood flow [25]. Myocardial blood flow (MBF) represents an integrated flow through both the macro and microcirculation (Figure 1). Thus, the assessment of MBF, via dynamic imaging modalities, provides the basis of CMD assessment. In the absence of obstructive epicardial disease, any reduction in the MBF is assumed to be caused by disruption in the coronary microcirculation causing CMD.

In clinical practice, non-invasive measurement of CMD relies on the principle of the coronary flow reserve (CFR). CFR represents the increase in coronary blood flow (CBF) that is achieved in going from basal coronary perfusion to maximal coronary vasodilation in response to a vasodilatory stimulus [26]. In patients with CAD, the extent of the reduction in CFR is directly related to the severity of stenosis, whereas in those with angiographically normal arteries, it is a marker of microvascular dysfunction. Thus, CFR does not differentiate epicardial from microvascular disease per se, but once angiography shows an absence of stenosis, it can be used as an index of CMD.

CFR is determined by taking measurements of coronary blood flow both at rest (basal flow) and with maximal hyperemia achieved by pharmacological/vasodilator stress. CFR is then expressed as the ratio of blood flow during hyperemia to the blood flow at rest [6,9,12,27,28]. There is no unified range for a normal CFR. This is because CFR measurements are affected by age, sex, the method used for its determination, rate–pressure product, unrecognized cardiovascular risk factors in supposedly healthy individuals, and test–retest variability [1,22,29]. A coronary flow reserve of less than 2.0 is often considered abnormal. Stress MBF is usually measured in day to day clinical practice via either PET or CMR. If an institution does not have either of these modalities, one can employ dynamic SPECT acquisition as described using solid state detectors, or MCE. Both of these are less sensitive than the two aforementioned techniques, but can be coupled with a diagnostic history to establish a clinical diagnosis.

The reference standard for measurement of CFR and hence diagnosis of CMD used to be invasive coronary reactivity testing (CRT). This involves the use of vasoactive substances to test endothelial and nonendothelial-dependent coronary function. CMD measured by CRT predicts adverse events in both men and women with and without obstructive CAD [3,22]. Standard noninvasive imaging techniques (stress echo and myocardial perfusion SPECT) are not reliable sources for the estimation of MBF and hence CFR and are often normal in CMD. Advancements in cardiovascular imaging techniques have revolutionized the noninvasive detection of CMD. Another concept in the evaluation of epicardial stenosis concerns the growing clinical application of fractional flow reserve (FFR). FFR is a pressure-derived index, expressed as a ratio of the maximal achievable CBF in the presence of an epicardial coronary stenosis to the maximum achievable flow if that artery were normal. This can be measured using a pressure wire in the coronary artery after administration of a vasodilator such as adenosine. In this case, pressure is used as a surrogate for flow using Poiseuille’s formula. Thus, FFR is calculated as the ratio of mean pressure distal (Pd) to a stenosis to the pressure proximal to the stenosis i.e., the aortic pressure, Pa, during maximal hyperemia. In stable coronary artery disease (CAD), the evidence base supports revascularization of lesions with an FFR of ≤0.80, whereas CAD associated with an FFR of >0.80 can be managed medically. The FAME-2 trial showed that FFR-guided PCI with drug-eluting stents plus medical therapy, as compared with the medical therapy alone, resulted in significantly improved clinical outcomes among patients with functionally significant stenoses and stable coronary artery disease [30]. The difference between the two strategies was driven by an increase in the need for urgent revascularization in the medical-therapy group. Computation fluid dynamics have now made it possible to assess FFR noninvasively using cardiac CT—this technique, called FFR_CT_, relies on myocardial segmentation and simulated flow using Navier-Stokes equations and correlates well with invasively measured values.

## 6. Review of Non-Invasive Modalities for CMD Assessment

### 6.1. Echocardiography

Exercise stress or dobutamine contrast echocardiography can detect ischemia in the presence of a hemodynamically significant epicardial stenosis; however, it has limited utility in the diagnosis of CMD if an ultrasound contrast agent is not employed. The reason for this is that CMD usually produces subendocardial ischemia, whereas gross hypokinesis by qualitative visual examination, as is done frequently for large vessel stenosis, requires a greater amount of transmural ischemic burden in the myocardial segments subtended by the stenotic artery. Similarly, patchy CMD would not be detected. In addition, there can be significant inter-observer variability in the interpretation of mild localized hypokinesia and hence results are not very reproducible [31,32]. The use of echocardiography to detect CMD relies mainly on myocardial contrast echocardiography (MCE), which is a low-risk bedside and inexpensive procedure to quantify MBF. Calculation of MBF, during a constant venous infusion of microbubble contrast, depends on the microvascular cross-sectional area as well as the mean velocity of microbubbles [33]. The rate of reappearance of microbubbles after destruction with ultrasound waves gives the mean velocity, whereas measuring their concentration in the myocardium provides the cross-sectional area [34]. Vogel et al. validated this method against PET with a correlation coefficient of 0.88 when measuring MBF in healthy volunteers [32]. Coronary flow velocity reserve (CFVR), using pulsed-wave Doppler sampling of the proximal left anterior descending coronary artery, provides another echocardiographic method of MBF assessment [35,36,37]. It is the ratio of CFV at stress to rest. CFVR correlates well with flow acquired from an intracoronary Doppler wire [35,38] and was able to identify a high prevalence of CMD in patients with heart failure with preserved ejection fraction in the PROMIS-HFpEF trial [39,40]. However, this technique correlated poorly with the gold standard, PET-estimated MPR, in the iPOWER study [31]. Echocardiographic assessment of CMD is limited since MBF assessment using contrast or CFVR is operator-dependent. Image quality is affected by artifacts, particularly in obese and patients with lung disease. Difficulties in post-processing due to movement of the imaging frame during the replenishment of microbubbles also poses a challenge. The use of microbubbles for myocardial perfusion assessment is currently not reimbursed in the United States, further hampering its clinical adoption. Nevertheless, it can provide an initial, inexpensive and crude assessment of microvascular perfusion and CMD.

### 6.2. Computerized Tomographic (CT) Angiography

**Figure 2:**
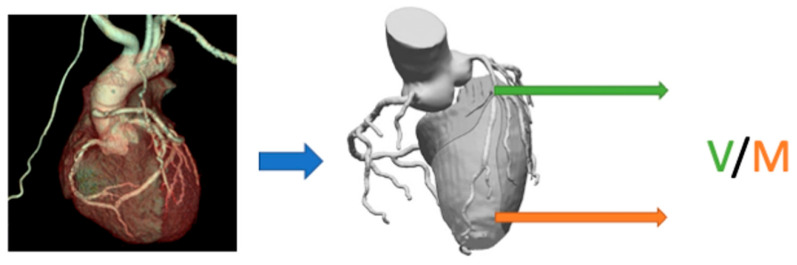
Calculation of V/M ratio from coronary computed tomography angiography (CTA) involves computation of the coronary volume (V, shown in green) and myocardial mass (M, shown in orange). A ratio of <2 denotes coronary microvascular dysfunction (CMD) in the absence of significant epicardial stenosis.

The advent of multi-detector dynamic CT imaging has improved the capability of CT to go beyond anatomic assessment of coronary luminal stenosis alone. Newer technological advances such as 256 and 320 row detectors allow for single heartbeat acquisition, which permits serial myocardial and left ventricular cavity sampling for quantifying the MBF [41]. Iodinated contrast is injected with prospective ECG triggering and scanning is performed every other RR interval to follow the first pass of contrast through the myocardium at frequent intervals. Mathematical modeling is then applied to these data to produce estimates of absolute myocardial flow. Various modeling techniques can be used, such as arterial input function, upslope analysis, or model-based deconvolution. Current state-of-the-art CT scanners have a spatial resolution of 0.5 mm that permits one to track the difference in attenuation between the endocardium and the epicardium. This delta gradient can be used to model MBF via the microcirculation. Patients with CMD will have a lower ratio of endocardial attenuation values to that of epicardial attenuation values, signifying reduced subendocardial perfusion. This technique relies heavily on adequate spatial resolution and a lack of beam hardening artifacts which are not just theoretical concerns, and thus it is not widely used in clinical practice currently. Furthermore, thinner myocardial wall thickness as in dilated cardiomyopathy can make transmural gradients of attenuation difficult to appreciate. Lastly, the rapid flow of contrast medium via the microcirculation itself can produce transient endothelial functional changes as well as epicardial vasodilatation, all of which can confound the normal physiology [6,42,43]. Coronary anatomic and myocardial perfusion assessment can be obtained using CT angiography (CTA) in combination with CT perfusion (CTP) [44]. CTA-derived FFR (FFR_CT_) is a sensitive tool for hemodynamic evaluation of lesion specific ischemia. It involves quantification of MBF and FFR_CT_ at a specified point in the coronary vasculature through computational fluid dynamics and uses a 3-D derived coronary model that simulates maximal hyperemia. The relationship between FFR_CT_ and CMD is not fully defined yet [12,43,45], but Nørgaard et al. showed that a low ratio of CTA-derived coronary luminal volume to myocardial mass was an independent predictor of ischemia in nonobstructive coronary disease [46]. Grover and colleagues expanded on the same concept and used coronary CTA derived mean total coronary luminal volume (V) and the mean myocardial mass (M) to compute the V/M ratio (Figure 2). The mean ratio of coronary luminal volume to myocardial mass (V/M) was significantly lower in the CMD group (25.6 ± 5.9 mm^3^/g vs. 30.0 ± 6.5 mm^3^/g; *p* = 0.007) [47]. The major advantages of this method are the superior spatial resolution of CT and rapid scan time. It also provides the opportunity to perform accurate anatomical and functional assessment of both the myocardium and the coronary circulation within one examination. Even though this technology can potentially identify CMD, CTA has limitations and is thus not routinely used for CMD assessment—some limitations include radiation exposure, caution regarding the use of iodinated contrast agents in renal insufficiency, and the potential for contrast-mediated vasodilation to overestimate the MBF [48,49].

### 6.3. Single-Photon Emission Computed Tomography

SPECT imaging has had limited diagnostic utility in the past for assessment of CMD, largely because of pharmacokinetics of the radiotracers used for SPECT myocardial imaging. The most commonly used technetium-based SPECT perfusion radiotracers (^99m^Tc-sestamibi, ^99m^Tc-tetrofosmin and ^99m^ Tc-teboroxime) have relatively low first-pass extraction, show significant roll-off of radiotracer uptake at higher flow rates, and have significant liver or intestinal uptake. This, along with poor camera sensitivity and temporal resolution (with the common sodium-iodide cameras), accounts for the suboptimal quantification of MBF [6,29].

However, with the recent advent of high-sensitivity cardiac cameras, iodinated rotenone compounds (^123^I-ZIROT,^123^I-CMICE-013) and solid-state high sensitivity cadmium-zinc-telluride detectors (CZT), dynamic SPECT can be used for quantification of MBF and hence for the assessment of CMD [6,50]. Agostini et al. assessed the feasibility of MBF and MFR estimation using dynamic SPECT in the prospective WATERDAY study. They validated dynamic CZT-SPECT (against ^15^O–water PET) as having a high diagnostic value for detecting impaired MFR in patients with stable CAD [50].

Dynamic SPECT protocols for MBF assessment might not achieve the accuracy and robustness of PET imaging, but could allow clinically useful measurements of MFR in a larger number of sites that do not have access to PET, and at a lower cost. Multi-center studies with larger cohort of patients are needed to confirm the robustness and reproducibility of these SPECT-derived measurements.

### 6.4. Positron Emission Tomography (PET)

PET provides global and regional measurements of perfusion, quantitative MBF and function, both at stress and rest, in a single examination. Quantification of MBF has been extensively validated with PET and it is the most widely used noninvasive modality for the clinical assessment of CMD [51]. Dynamic first pass PET scanning provides accurate MBF measurements. It involves the use of post-processing software that performs automated segmentation and arterial input function measurements to compute the regional and global stress and rest MBF (in milliliters of blood per minute per gram of myocardium) [25,52,53].

The commonly used PET radiotracers are ^13^N-ammonia, ^82^Rb, and ^15^O-water. ^82^Rb is the most commonly used radiotracer because it requires only an on-site generator as opposed to a cyclotron [54]. It has the disadvantage of low extraction fraction, significant roll-off at high coronary flows, and higher radiation as compared to ^13^N-ammonia and ^15^O-water, which are better agents for MBF estimation considering higher first pass uptake (100% for ^15^O-water) and minimal roll-off. However, the latter 2 are not as readily available for day-to-day clinical use.

PET has been validated against invasive modalities of flow estimation in numerous studies. Absolute quantification of MBF and CFR is highly reproducible over a wide range of MBF (0.5–6 mL/g per minute) [6]. PET can also simultaneously assess all three coronary distributions, thus allowing for a more accurate assessment of CMD, which can have heterogeneous regional myocardial distribution [55].

**Figure 3:**
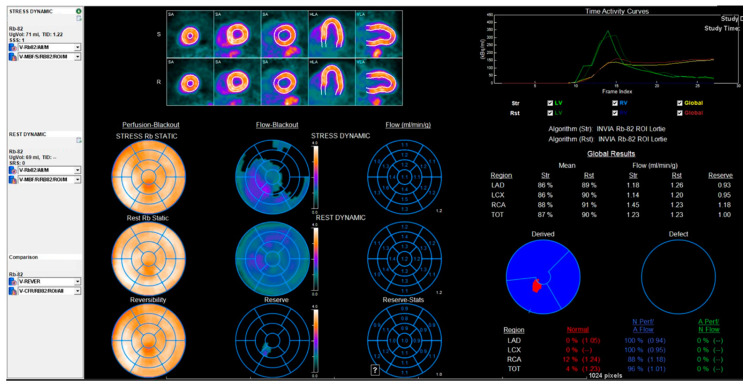
Rubidium-82 regadenoson stress-rest PET (positron emission tomography) in a 64-year-old man with persistent, recurring angina and only mild epicardial CAD (coronary artery disease) on invasive angiography done at an outside hospital. There is abnormal global myocardial perfusion reserve (MPR) <2 consistent with CMD (coronary microvascular dysfunction). He was prescribed Ranolazine and high intensity statin therapy, and had dramatic improvement in 1 months’ time. LAD = Left anterior descending coronary artery, RCA = Right coronary artery, LCX = Left circumflex artery, TOT = Total, LV = Left ventricle, RV = Right ventricle.

CMD detected by PET as abnormal myocardial perfusion reserve (MPR) has also been correlated with adverse outcomes (Figure 3). Ziadi et al. reported that MPR quantified using ^82^Rb PET predicted hard cardiac events independent of the summed stress score and other parameters. They also suggested that routine assessment of ^82^Rb PET–quantified MBF and MPR could improve risk stratification for patients being investigated for ischemia. PET has also been used to validate CMD seen in metabolic syndrome patients [56]. These findings are particularly profound in women, indicating important sex-related differences in the pathogenesis of CMD [1,15,19,22]. Despite the diagnostic and well validated prognostic data in PET-derived MBF/CFR, it is not without its limitations. It entails high radiation exposure and cost, depending on which radiotracer is used [57].

### 6.5. Cardiac MRI

Cardiac magnetic resonance (CMR) is an ideal noninvasive modality for assessing patients with microvascular angina/CMD. Two types of stress CMR modalities have been tested—stress perfusion and stress T1 mapping.

Stress CMR perfusion can detect myocardial perfusion abnormalities with high spatial resolution without exposure to ionizing radiation. The qualitative evaluation is done by the visual analysis of first-pass gadolinium images [58,59]. This approach does provide a reliable diagnostic tool for single or two-vessel CAD. However, visual assessments can be less accurate when MBF is globally reduced, i.e., in three-vessel disease or in CMD. It also lacks reliable quantification of the severity of anatomic stenosis or distinguishing diffuse three-vessel disease from CMD.

**Figure 4:**
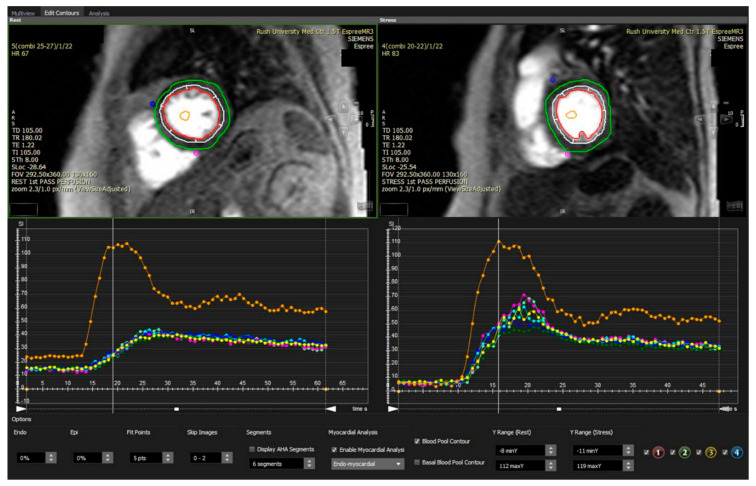
Stress CMR (Cardiovascular Magnetic Resonance) perfusion in a 67-year-old woman with angina and negative coronary CTA (computed tomography angiography) that had shown minimal calcific plaque with <10% stenosis in the proximal left anterior descending artery. After regadenoson stress, no visual reversible perfusion defect was noted, indicating the absence of ischemia from epicardial stenosis; however, the MPRI (myocardial perfusion reserve index) is computed at 1.97, consistent with the diagnosis of CMD (coronary microvascular dysfunction). She was initiated on amlodipine, a high intensity statin and long-acting nitrate therapy and had resolution of anginal symptoms after 2 weeks. In the top panel, Green contours depict epicardium of the left ventricle, Red depicts the endocardial border, and White is the mid myocardium. In the bottom panel, the Orange curve is the signal intensity of blood pool in the left ventricle plotted against time—during Stress regadenoson imaging on the right, and at Rest on the left. The other colored curves depict various segments of the left ventricular myocardium.

Quantitative and semi-quantitative evaluation of the first-pass perfusion images can be used to calculate objective parameters of perfusion, such as the global stress myocardial flow reserve (SMFR) and the myocardial perfusion reserve index (MPRI), which is an indexed ratio of perfusion-time intensity curve upslopes in response to vasodilator stress (Figure 4). Other, more complex approaches focus on the detection of diffuse myocardial fibrosis, noninvasive measurement of cellular oxygenation, or the detection of early abnormalities in diastolic filling [60].

**Figure 5:**
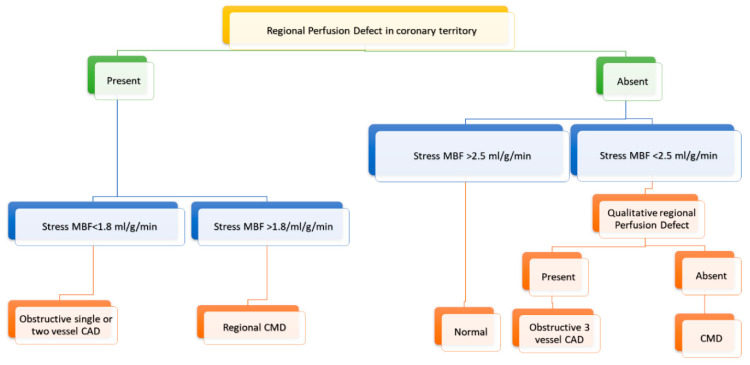
Diagnostic algorithm at Rush University Medical Center for detection of obstructive CAD (coronary artery disease) and coronary microvascular dysfunction (CMD) using regadenoson stress CMR (cardiovascular magnetic resonance) perfusion. MBF, Myocardial blood flow.

Kotecha et al. showed that an automated pixel-wise quantitative myocardial perfusion mapping technique can be used to detect and differentiate CMD from three-vessel disease with good accuracy. They suggested an algorithm for the detection of obstructive coronary artery disease and CMD based on regional and global stress MBF (Figure 3). Patients with global stress MBF < 2.25 mL/g/min without visual defects are likely to have CMD. In comparison, patients with regional perfusion defect and regional stress MBF <1.94 mL/g/min were likely to have obstructive one- or two-vessel disease. Global stress MBF <2.25 mL/g/min with visual perfusion defects was likely to be obstructive three-vessel disease (Figure 5) [61].

Another novel CMR technique, known as T1 mapping, represents a breakthrough in the non-invasive assessment of microvascular ischemia when performed during vasodilatory stress [62,63]. It has the ability to noninvasively diagnose and differentiate between epicardial CAD and CMD using T1 relaxation time without the use of gadolinium contrast. T1 represents the exponential recovery of the longitudinal component of magnetization (Mz) back towards its thermal equilibrium and is a fundamental magnetic resonance property. It is influenced by intrinsic tissue properties, the extrinsic tissue environment, as well as the hardware and software used for the measurement [63]. T1 mapping displays the T1 values of imaged tissues on a voxel-by-voxel basis, enabling quantitative myocardial tissue characterization [64]. T1 predominantly detects free water, and increased free water content in tissue, such as edema or water collecting in the expanded interstitial space and hence native myocardial T1 time is prolonged with increased free water content [65,66].

Assessment of the coronary vasculature using T1 mapping relies on the principle of coronary vasodilatory reserve and myocardial blood volume (MBV). In healthy myocardium and coronary vasculature, MBV may increase two-fold during coronary vasodilatory stress, representing significant coronary reserve. This corresponds to a 6% increase in myocardial T1 during adenosine vasodilatory stress [62]. In patients with obstructive CAD, the coronary vasodilatory reserve is diminished, because the microcirculation in resting ischemic myocardium downstream from the significant stenosis undergoes compensatory vasodilation to maintain myocardial perfusion [67,68]. This process increases the resting MBV and hence the free water content in the ischemic myocardium, which is detectable by using T1 mapping. Liu et al. verified these findings by showing that the myocardium downstream of non-obstructive coronary arteries had normal resting T1 with an increase of >4% during stress. Myocardial territories downstream of obstructive epicardial CAD (i.e., those with invasive fractional flow reserve of <0.8) had an elevated resting T1, which augmented minimally with adenosine stress, leading to a near-zero stress T1 response (delta T1 < 1.5%). The presence of CMD (FFR > 0.8 and index of microvascular resistance >25 units) was characterized by a blunted but detectable stress T1 response (delta T1 1.5 to 4%) compared with myocardium downstream of obstructive epicardial CAD [64].

The difference in diagnostic performances of perfusion CMR and stress T1 mapping is due to different mechanisms for ischemia detection as described above. Hence, although MBF, as assessed quantitatively by perfusion CMR, is similar downstream of obstructive and nonobstructive coronary arteries, there is a significant observed difference in resting myocardial T1.

Arterial spin labeling (ASL) is an emerging technique that has been used successfully in non-cardiac MR modalities in the past to measure regional cerebral, renal and skeletal muscle blood flow [69,70,71]. In ASL, there is a magnetic tag given to the protons in arterial blood. This is different in magnetization from that of the surrounding soft tissue. Once a ‘control’ scan is obtained without tagging of the arterial blood, we can measure the signal difference of the tagged blood flow from the untagged image. The difference between these two images reflects the amount of tagged blood that has been delivered to the imaging region and, with appropriate tagging and imaging methods, reflects local tissue blood flow [72,73]. Zun et al. showed that ASL CMR detects a clinically relevant increase in MBF with vasodilation and these measurements were consistent with ranges established by quantitative PET [73]. Currently, ASL is in the technological developmental stage and holds future promise in detection of CMD.

More recently, arterial blood deoxygenation techniques with hyperventilation have been used to detect CMD in heart transplant recipients using oxygen sensitive CMR sequences. CMD can occur even in the absence of overt allograft vasculopathy and increases the risk of adverse cardiac outcomes [74].

Non-contrast-based CMR techniques are the future of CMR-derived CMD assessment. Besides higher diagnostic performance, they have the potential to address the limitations of gadolinium-based MBF assessments including imaging artifacts, long scan time, interobserver variability, problems with the absolute quantitation of MBF, restricted use in chronic kidney disease patients and a lack of widespread availability of quantitative first-pass sequences [29].

## 7. Treatment

The multifactorial pathophysiology and varied phenotypes of CMD makes it a challenging clinical entity to manage. Treatment thus far has been empirical and involves cardiovascular disease (CVD) risk factor modification and conventional pharmacological options for obstructive atherosclerotic CAD. Lifestyle modification with smoking cessation, weight loss, regular exercise and improved nutrition along with the optimization of underlying hypertension, diabetes mellitus and dyslipidemia has been shown to improve overall ischemic burden by improving microvascular circulation [15].

Pharmacological therapies to target microvascular angina can be chosen based on specific CMD phenotype. Antiplatelets and lipid lowering drugs are the standard of care because of the strong association of atherosclerosis and CMD. Statins with evident mortality benefit in obstructive CAD have been shown to improve microvascular perfusion by increasing CFR [75]. Symptomatic management of involves use of anti-anginal agents. Beta-blockers and short acting nitrates can relieve symptoms of microvascular angina. In cases where coronary vasospasm is suspected to be the predominant pathophysiological mechanism, calcium channel blockers and long-acting nitrates can provide symptom relief. Angiotensin-converting enzyme inhibitors and angiotensin receptor blockers can prove helpful by improving microvascular perfusion. There is a theoretical benefit of using anti-anginal agents such as ranolazine in refractory cases of CMD-mediated angina. However, recent studies have shown no significant improvement in symptoms or change in coronary microvascular function in patients with CMD [76,77]. Surgical management is limited to weight reduction surgery targeting the CMD phenotype in morbidly obese patients and has been shown to improve microvascular function [78]. Novel therapies in trials are targeting the role of anti-inflammatory drugs and endothelial vasodilators. The UMPIRE trial was designed to evaluate the effect of udenafil, a phosphodiesterase-5 inhibitor, in female patients with CMD. It aims to study the effect of udenafil on CMD symptoms as well as perfusion defect size using adenosine-stress CMR [79].

## 8. Conclusions

A number of validated, noninvasive cardiovascular imaging modalities are presently available to measure MBF and CFR in the setting of CMD (Table 1 summarizes the strengths and weaknesses of non-invasive imaging modalities for CMD diagnosis). Quantifiable end points and algorithms have been developed for the diagnosis of CMD and will be helpful for future studies.

PET is currently the most studied and validated modality, but CMR-derived MBF and T1-mapping measures are being increasingly used to diagnose patients and assess their response to treatment. In clinical practice, the type of imaging modality used is dependent on individual risk–benefit ratio and cost, in addition to local expertise and availability of the technology. Additional studies are currently examining integrative imaging approaches and regional versus global MPR assessments. The ultimate goal of using these modalities will be to define prognostic differences, therapeutic interventions, and treatment approaches, such that we can make an impact on the profound morbidity and non-negligible mortality associated with this condition. 

## Figures and Tables

**Table 1:** Strengths and limitations of the various common noninvasive modalities for the diagnosis of CMD.

Imaging Modality	Availability	Accuracy	Reproducibility	Prognostic Validation	Diagnostic Parameter	Cost
Echocardiography	++++	++	+++	+++	CFVR	$
Cardiac CT	+++	+	+	n/a	V/M	$$
SPECT	++++	+	++	+	CFR	$$
PET	++	++++	++++	++++	CFR	$$$
CMR	++	+++	+++	++	MPRI	$$$

CFVR = coronary flow velocity ratio, V/M = coronary volume-to-myocardial mass ratio, CFR = coronary flow reserve, MPRI = myocardial perfusion reserve index, CT = computed tomography, SPECT = single photon emission computed tomography, PET = positron emission tomography, and CMR = cardiovascular magnetic resonance. The plus symbols (+) refer to the degree to which that imaging modality fulfills the attribute listed in the column. The dollar ($) signs refer to relative expense.
